# Construction and Preliminary Characterization Analysis of Wuzhishan Miniature Pig Bacterial Artificial Chromosome Library with Approximately 8-Fold Genome Equivalent Coverage

**DOI:** 10.1155/2013/587493

**Published:** 2013-04-18

**Authors:** Changqing Liu, Yuo Guo, Taofeng Lu, Hongmei Wu, Risu Na, Xiangchen Li, Weijun Guan, Yuehui Ma

**Affiliations:** ^1^Institute of Beijing Animal Science and Veterinary, Chinese Academy of Agricultural Science, Beijing 100194, China; ^2^Bioscience Department, Department of Laboratory Medicine, Bengbu Medical College, Bengbu 233030, China

## Abstract

Bacterial artificial chromosome (BAC) libraries have been invaluable tools for the genome-wide genetic dissection of complex organisms. Here, we report the construction and characterization of a high-redundancy BAC library from a very valuable pig breed in China, Wuzhishan miniature pig (*Sus scrofa*), using its blood cells and fibroblasts, respectively. The library contains approximately 153,600 clones ordered in 40 superpools of 10 × 384-deep well microplates. The average insert size of BAC clones was estimated to be 152.3 kb, representing approximately 7.68 genome equivalents of the porcine haploid genome and a 99.93% statistical probability of obtaining at least one clone containing a unique DNA sequence in the library. 19 pairs of microsatellite marker primers covering porcine chromosomes were used for screening the BAC library, which showed that each of these markers was positive in the library; the positive clone number was 2 to 9, and the average number was 7.89, which was consistent with 7.68-fold coverage of the porcine genome. And there were no significant differences of genomic BAC library from blood cells and fibroblast cells. Therefore, we identified 19 microsatellite markers that could potentially be used as genetic markers. As a result, this BAC library will serve as a valuable resource for gene identification, physical mapping, and comparative genomics and large-scale genome sequencing in the porcine.

## 1. Introduction

The availability of large-insert genomic DNA libraries is essential for physical analysis of large chromosomal regions, map-based gene isolation, and gene structure and function analysis. It also has been considered a powerful approach complementary to experimentation that helps to identify functional elements and evolutionary constraints within the genomes [1, 2]. Currently, the bacterial artificial chromosome (BAC) library is the common choice of resources for cloning of genes of unknown sequences by chromosome walking. It provides easy access to stable material for DNA manipulation such as exon trapping, cDNA selection, direct sequencing, microsatellite marker isolation, fluorescence in situ hybridization (FISH), and physical mapping [3–5].

BAC libraries have been constructed for several species of agricultural importance, including cattle [6], bovine [7], goat [8], horse [9], pig [10–12], and sheep [13]. Large-insert DNA libraries from pig have been used for in situ hybridization experiments, whole-genome physical mapping and isolation of genes and gene clusters as well as for the identification of regulatory elements. 

Wuzhishan miniature pig is one of the four most important pig breeds in China, has many major economic characteristics such as high rate of production of meat and meat quality, enjoyed a high international reputation, and is considered useful for medical and veterinary research due to its small size. In 2006, the Chinese government brought it into 138 state-level protected domestic animal breeds. Here, we describe the construction and characterization of Wuzhishan miniature pig BAC library from blood cells and fibroblasts, respectively. Additionally, 19 microsatellite markers and SRY gene were used to screening the BAC library. We expect this key resource will facilitate further genetic studies in this important species.

## 2. Materials and Methods 

### 2.1. Preparation of BAC Vector

The BAC vector pBeloBAC11, provided by New England Biolabs, was isolated using Qiagen Plasmid Mega Kit (Qiagen, Valencia, CA, USA) following the published protocol [14]. To obtain pure circular plasmid DNA, the vectors were further subject to a CsCl-ethidium bromide density gradient centrifugation. After establishing the minimal amount of restriction enzyme required for complete digestion of the vector in a series of test reactions, the vector DNA was digested with an appropriate amount of *Hind* III restriction endonuclease (New England Biolabs) and treated with alkaline phosphatase (CIAP) for dephosphorylation [15]. Linear vectors were recovered by electroelution to remove nondephosphorylated linear vectors.

### 2.2. High-Molecular-Weight (HMW) Genomic DNA Preparation

The fibroblast cell line of six male Wuzhishan miniature pigs was obtained using primary explanting technique and cell cryogenic preservation technology. Characteristic tests for established cell line with cell viability, microorganism detection, and chromosome analysis were performed as described by Talbot et al. [16]. White blood cells and fibroblasts from an adult male Wuzhishan miniature pig were collected independently and mixed with 1% Seaplaque GTG agarose (Cambrex) at a concentration of 5 × 10^7^ cells/mL. The cell-agarose suspension was transferred into DNA plug molds to form solid agarose plugs. The agarose plugs were treated with freshly prepared proteinase K and partially digested using *Hind* III as described in published protocols [17]. Sided by low-molecular-weight markers (New England Biolabs), the digested DNA plugs were subject to PFGE and the gel block containing large-size DNAs (100–400 kb) were cut in 0.5 cm slices. A second PFGE was then performed to remove small DNA fragments coiled within the large DNA fragments in the gel slices. Through electroelution and dialysis, the HMW DNAs were purified and quantified by agarose gel electrophoresis with the *λ* DNA marker of known concentration.

### 2.3. Ligation and Transformation

Diluted DNA was quantitated and ligated by adding the pBeloBAC11 vector in a molar ratio of 5–10 : 1 in a 50-uL reaction volume at 16°C overnight. The ligation mixture was then dialyzed on a microdialysis filters (0.025 mm pore size; Millipore) against 0.5 × TE for 60 min. After ligation and microdialysation, 2 uL of the dialysed ligation product was used to electrotransform 20 uL of an *Escherichia coli* Electro MAX DH10B competent cells (Invitrogen) in Gene Pulser apparatus (BTX-ECM630) at different voltages (~1.3–2.5 kV/cm) to maximize the transformation efficiency. After electroporation, the mixtures were shaking-incubated at 37°C for 1 h and then plated on Luria-Bertani (LB) agar plates (100 mm × 15 mm) containing 20 ug chloramphenicol/mL and incubated at 37°C for 16 h.

### 2.4. Large-Scale BAC Clone Production

The ligation was scaled up under optimized conditions, and the transformed cells from six electroporations were pooled together for large-scale production. The automatic colony picker (QPIX II, Genetix) was used to array the BAC clones into 384-well microtiter plates. The plates were incubated at 37°C for 14–16 h to ensure the proper growth of the clones for accurate picking. After picking, the plates were stored in −80°C freezers. The media containing 7% glycerol was used in the 384-well plates to protect the *E. coli* cells from damage under frozen conditions.

### 2.5. Insert Size Distribution of Wuzhishan Miniature Pig BAC Library

A total of 270 BAC clones (120 from blood cells and 150 from fibroblast cells) were randomly picked from the Wuzhishan miniature pig library. These clones were incubated in 10 mL Luria-Bertani (LB) medium containing 20 ug chloramphenicol/mL at 37°C for 16 h. The BAC DNAs were isolated using a rapid alkaline lysis miniprep method and digested individually at 37°C for 3 h with 0.5 U *Not *I. The molecular weights of the BAC inserts were calculated according to the low-range size marker by PFG electrophoresis.

### 2.6. Library Pooling and BAC Library Screening

To establish the two-step PCR screening systems, the library was divided into 20 superpools and one superpool comprised 20 384-well plates. Cultures from every superpool were combined to make superpool DNA for the first step PCR screening. In each superpool, cultures from each plate (384 clones), row (24 clones × 20 plates), and column (16 clones × 20 plates) were combined, respectively, to make DNA for the second step screening. Primer sequences for 19 microsatellites selected from different regions of swine genome and for SRY gene were designed according to the swine DNA sequences published in NCBI. BAC screening was performed by two-step PCR (superpools PCR and 4D-PCR) [18]. Positive BAC clones were confirmed by the fragments which were amplified for the expected size and sequencing of PCR products.

## 3. Results

### 3.1. Cell Cultures and Characteristic Tests

**Figure 1:**
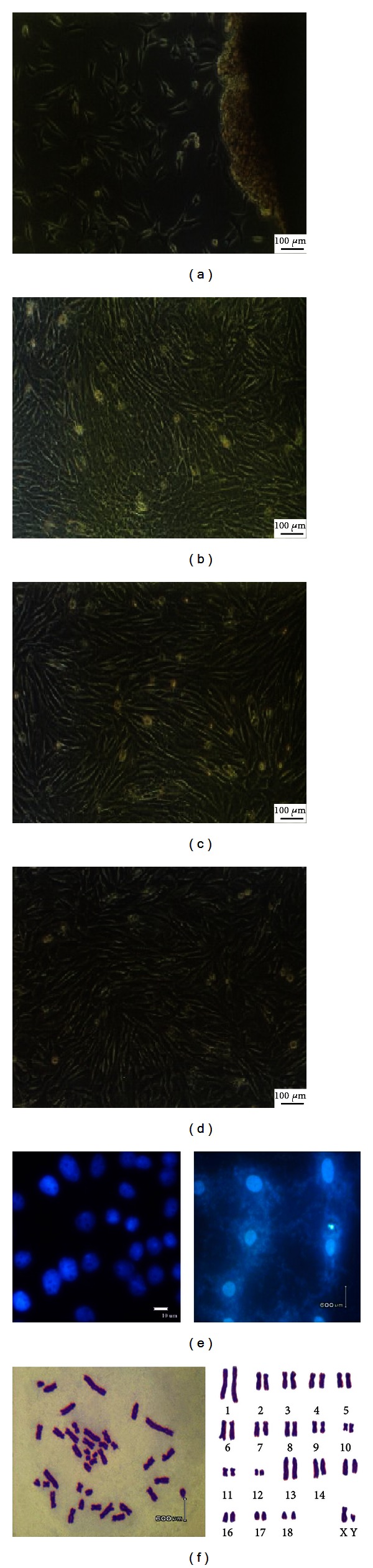
Morphology, mycoplasma contamination, and karyotype of Wuzhishan miniature pig cell line. (a) Primary cells (×100), the cells were typical long spindle-shape. (b) Subcultured cells. (c) Cells before cryopreservation. (d) Cells after recovery. (e) Mycoplasma test stained with Hoechst33258 and positive control of mycoplasma contamination; (f) chromosome at metaphase (left) and karyotype (right) (×1,000).

We used primary explanting technique and cell cryogenic preservation technology to establish the Wuzhishan miniature pig fibroblast cell line and proceeded to biological and genetic detection. The culture conditions were optimal, and the cells were healthy (Figures 1(a)–1(d)). Because we want to construct the BAC library to conserve genomic character of Wuzhishan miniature pig, the fibroblast must maintain diploid character similar with the cells *in vivo*. Chromosome analysis showed that the frequency of cell chromosome number of 2*n* = 38 was 90.4–92.6% in passage 1 to 3, which indicated culture *in vitro* effect the heritage of cells slightly, supporting that the cell line was a steady diploid one (Figure 1(f)). The test results of the bacteria, virus, and mycoplasma were negative (Figure 1(e)). 

### 3.2. Vector Preparation and High-Molecular-Weight (HMW) Genomic DNA Preparation

**Figure 2:**
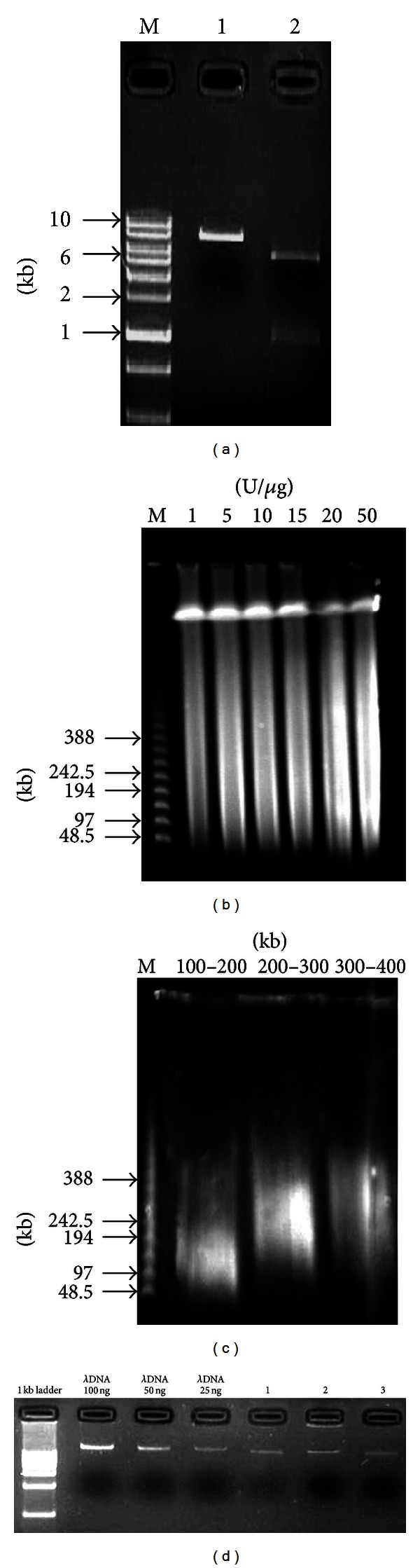
Characterization of digested BAC vector and PFGE size fractionation of digested HMW by *Hind* III. (a) Characterization of digested vector by electrophoresis; M: 1 kb DNA ladder; 1: BAC vector digested by *Hind* III; 2: BAC vector digested by B*am*H I and X*ho* I; (b) initial PFGE size fractionation of partially digested HMW; M: Lamda Ladder PFG Marker; 1, 5, 10, 15, 20, and 50 U/*μ*gDNA, respectively; (c) second-size selection PFGE, used to eliminate smaller DNA molecules; M: Lamda Ladder PFG Marker; 100–200 kb, 200–300 kb, 300–400 kb, respectively; (d) quantification of Wuzhishan miniature pig genomic DNA.1: 100–200 kb DNA; 2: 200–300 kb DNA; 3: 300–400 kb DNA.

The most important step in constructing BAC library was isolation and partial digestion of high-molecular-weight DNA. Partial restriction enzymes digests were therefore assessed by monitoring the appearance of DNA smaller than 100 kb and the decrease in DNA in the high-molecular-weight (>1 Mbp) condensed zone (Figures 2(a), 2(b), and 2(c)). Select the insert DNA solution with the greatest concentration of high molecular weight DNA for use in subsequent ligation reactions. In our experience, the 100–200 kb, 200–300 kb, and 300–400 kb solutions have DNA concentration >5.0 ng/*μ*L can be used in ligation (Figure 2(d)). If the insert solutions are particularly dilute (<5.0 ng/*μ*L), they can be concentrated using Millipore nitrocellulose filters and 10% PEG High MW (insert) DNA samples are quite unstable. Though they can usually be left at 4°C overnight, it is best to perform ligation immediately after checking the DNA concentration.

### 3.3. Characterization and Insert Size Testing of BAC Library

**Figure 3:**
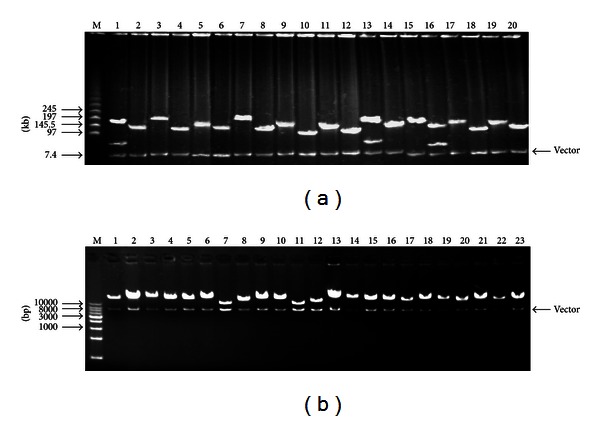
Characterization and Insert size testing of Wuzhishan miniature pig BAC Library. (a) Analysis of the size of BAC clones by PFG electrophoresis. M: Ladder PFG Marker; Lanes 1–20: randomly picked recombinant BAC DNA digested with *Not *I; (b) Analysis of the size of BAC clones by GEL electrophoresis. M: 1 kb DNA ladder plus; Lanes 1–23: randomly picked recombinant BAC DNA digested with *Not *I.

**Figure 4:**
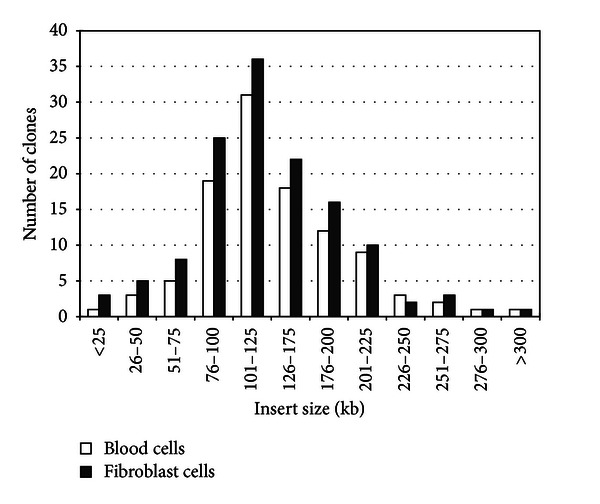
BAC insert sizes distribution in the library. Insert sizes were determined from 270 BAC clones. The horizontal axis shows the size range in kb while the vertical axis displays the number of clones corresponding to each size range. Insert sizes are reported in a cumulative histogram.

The library contains approximately 153,600 clones, arrayed into 400 384-well microtiter plates using an automatic clony picker. Among these 153,600 clones, 92,160 were from blood cells and 61,440 from fibroblast cells (Table 1). A total of 270 BAC clones were randomly sampled. The extracted DNA was digested with *Not* I to determine the size of the pig genomic insert (Figure 3). The average insert size of BAC clones was estimated to be 152.3 kb, with the small inserts (50–80 kb) accounting for less than 4.5%, nonrecombinants only 1.5%, 84.8% of the inserts ranged from 80 kb to 200 kb, and 78.6% were more than 100 kb (Figure 4), which is significantly higher than the previous porcine genomic library [10–12]. Thus this library is approximately 7.68-fold genome equivalents with unbiased chromosomal distribution, representing a 99.93% statistical probability of obtaining at least one clone containing a unique DNA sequence in the library.

### 3.4. The Testing of Library Stability

**Figure 5:**
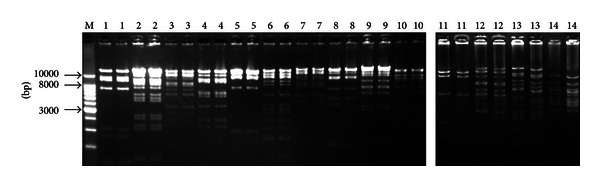
Examination of the BAC colony stability. M was 1 kb DNA Ladder; lanes 1–10 were restrictive finger print of different BAC clones from blood cell and 11–14 from fibroblast cells in second electrophoresis; every clone has two electrophoretic bands, the first was at day 1 and the second was at day 5.

To assess library stability, 14 random BAC clones were assayed by serial culture for more than 100 generations over a period of 5 days. The electrophoretic patterns of each clone digested with *Not* I were identical from days 1~5, confirming that the Wuzhishan miniature pig BAC-clone inserts were stable after long-term culture (Figure 5). 

### 3.5. BAC Library Screening

To estimate the genome coverage of the BAC library by determining the number of clones that contain selected DNA markers or known functional genes [11], the BAC library was screened by PCR for 19 microsatellite markers and SRY gene. Positive BACs for these markers and gene ranged from 2 to 16 with an average of 7.89 clones (Table 2), 4.58 from blood cells, and 3.31 from fibroblast cells. The average of 7.89 positive clones is compatible with the estimate of a 7.68-fold redundant library and confirmed that the library is unbiased. However, only SRY gene was not found in the library.

## 4. Discussion

In the last 15 years, BAC libraries have been extensively used in physical mapping and complete eukaryote genome sequencing [19–22]. Previous studies have shown that a clonal coverage of 6.0–8.0 genome equivalents was sufficient for the development of a genome-wide physical map of approximately 95% genome coverage. Wuzhishan miniature pig BAC library was constructed using blood cells and fibroblast cells from a male sample, respectively. The average insert size of Wuzhishan miniature pig BAC clones was estimated to be 152.3 kb, representing approximately 7.68 genome equivalents, which is significantly higher than the previous porcine genomic library, and we identified and validated 19 microsatellite markers that could potentially be used as genetic markers. The BAC library described here will be valuable for accurate assembly of the porcine genome sequences, the physical mapping of the pig genome, and the construction of minimum tiling paths for region-specific resequencing. 

The Wuzhishan miniature pig BAC library was constructed from male DNA; thus, both X and Y chromosomes are represented, but each sex chromosome is underrepresented because only one copy of each is present in the genome with 2 copies of every autosome. This characteristic must be considered when using the library to screen genes on the X and the Y chromosomes that are not part of the pseudo-autosomal region.

We have developed an optimal procedure for constructing the Wuzhishan miniature pig library effectively. Firstly, in order to recover the linearized and dephosphorylated vector by electroelution, we effectively reduced the background of transformation and optimized the ratio of vector and insert DNA in ligation. Secondly, we modified the protocol of HMW DNA preparation and made it suitable for pig species. Removing small restriction fragments is vital for construction of a high-quality BAC library [23]. Wuzhishan miniature pig genomic plugs prepared by the method used in this paper contained high quality and large quantity HMW DNA (about 10 *μ*g/plug), with no inhibitor, no mtDNA and very little small fragment contamination, produced satisfactory restriction fragments when digested with *Hind* III. Thirdly, to avoid contamination with small-trapped DNA fragments and improve the size and uniformity of the inserts, we performed two separate size selections.

Efficient library screening is crucial for all applications of the library. Screening can be performed either by hybridization on high-density filters or by the polymerase chain reaction (PCR) [24]. PCR screening, however, is much more reliable, faster, and efficient with higher specificity owing to effective avoidance of false positive clones identified by repeat sequences in probes by hybridization. Here, we used a four-step PCR screening procedure based on the BAC library pool system. The BAC pool strategy was sensitive enough to identify single positive clones among superpools containing 384 BAC clones. BAC clones cultured overnight served as PCR template directly, rather than using prepared BAC-DNA. This modification considerably simplifies the procedure and shortens the time required for library screening. Thus, we identified 19 microsatellite markers that could potentially be used as genetic markers.

## 5. Conclusions

The first high-quality, representative Wuzhishan miniature pig genomic BAC library had been constructed, covering about 7.68-fold genome equivalents of the porcine genome. There were no significant differences of genomic BAC library from blood cells and fibroblast cells. The availability of the Wuzhishan miniature pig BAC library will aid in identification of genes and genomic regions of interest, identify specific genes of interest, develop genetic markers, and for BAC end sequencing and deep sequencing of selected clones. 

## Figures and Tables

**Table 1:** Summary of the Wuzhishan miniature pig BAC library.

Segment	Sample	Cloning enzyme	Total clones	Plate No.	Non-insert clone (%)	Average insert size^a^ (kb)
1	Blood cells	*Hind* III	92160	1–240	0.8	164.4
2	Fibroblast cells	*Hind* III	61440	241–400	2.0	134.2

Total			153600	400	1.5	152.3

Note: Genome coverage 7.68× represented probability of finding a target gene was 99.93%. ^a^Determined using random clones from ligations.

**Table 2:** Summary of results from screening the Wuzhishan miniature pig BAC library by PCR with primers for microsatellites. The number of positive superpools for each locus (NF: not found) and the number of positive BAC clones are listed (Blood/Fibroblsats).

Marker name	Location Chr	Number of positive superpools	Number of positive clones
SW974	1	3/2	6/3
SW942	2	4/2	9/7
SW2429	3	2/2	5/5
SW1089	4	NF/2	0/2
SW378	5	3/2	6/3
SW1647	6	2/2	4/5
SW859	7	2/3	5/7
SW1080	8	3/2	6/2
SW911	9	1/NF	3/0
SW443	10	3/1	8/3
SW435	11	2/3	6/5
SW1553	12	NF/2	0/3
SW163	13	1/3	3/7
SW857	14	3/1	8/3
SW1416	15	2/3	2/3
SW2517	16	1/NF	3/0
SW1891	17	2/2	5/2
SW1682	18	NF	2/1
SW1411	X	2/1	8/3
SRY	Y	NF	0/0

Average number of positive clone			4.58/3.31
